# Surgical Excision of Multiple Penile Syringomas With Scrotal Flap Reconstruction

**Published:** 2014-06-11

**Authors:** Elbert E. Vaca, Gerhard S. Mundinger, Jonathan A. Zelken, Gulsun Erdag, Michele A. Manahan

**Affiliations:** ^a^Department of Plastic and Reconstructive Surgery, Division of Dermatopathology, Johns Hopkins Hospital, Baltimore, Md; ^b^Department of Dermatology, Division of Dermatopathology, Johns Hopkins Hospital, Baltimore, Md

**Keywords:** syringoma, penile syringoma, penile reconstruction, scrotal flaps, penis

## Abstract

**Objective:** Penile syringomas are rare lesions usually occurring in isolation. We report the excision and reconstruction of multiple synchronous penile shaft syringomas with local scrotal flaps. **Methods:** We report a rare case of excision of multiple penile syringomas and reconstruction with scrotal flaps in a 29-year-old man. **Results:** Penile syringomas were excised and reconstructed with scrotal flaps in a single-stage procedure. **Conclusions:** In addition to providing wound coverage, this reconstructive option allowed for excellent functional results with regard to shaft alignment and erectile function, and it should be considered in the reconstructive armamentarium for penile shaft lesions.

Syringomas are thought to be benign neoplasms of eccrine gland origin.^1^ Friedman and Butler^2^ proposed a classification system consisting of 4 principal clinical variants of syringoma: a localized form, a familial form, a form associated with Down's syndrome, and a generalized form that encompasses multiple eruptive syringomas. Some evidence suggests that eruptive forms represent reactive hyperplasia to insults such as waxing.^3^^,^^4^

Syringomas are more common in women and tend to appear during puberty or in early adulthood, most commonly localizing to the lower eyelid, but are also common on the upper cheek, upper chest, axilla, abdomen, and vulva.^5^^-^7 Penile involvement is rare—only 12 cases have been reported to date—with a tendency to present as isolated lesions during early adolescence, most commonly located over the base of the shaft.^5^^,^^8^^-^17 We report the first described surgical excision and reconstruction of multiple penile syringomas.

## METHODS

**Figure 1:**
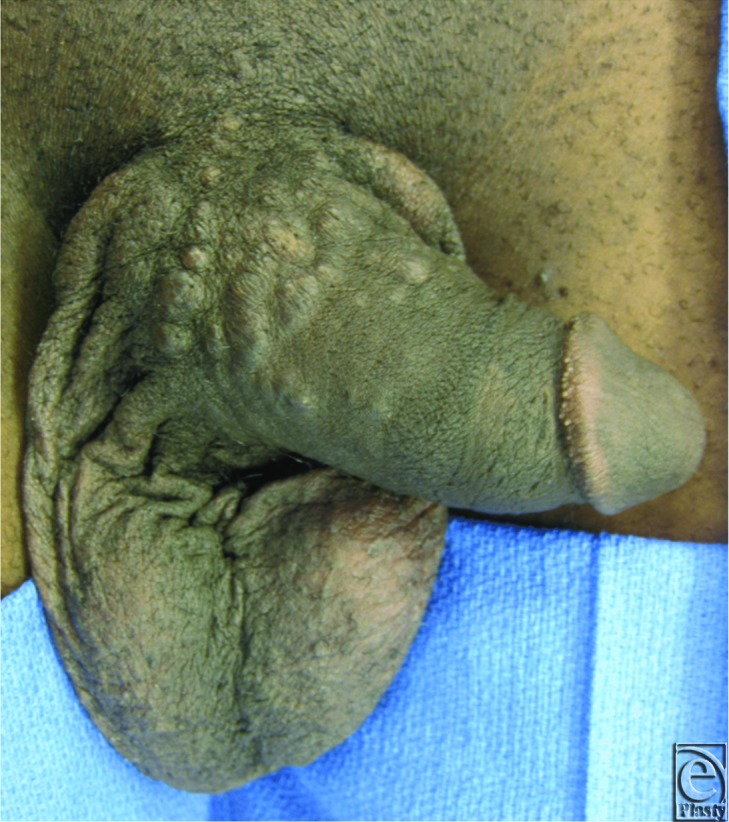
Multiple syringomas on the penile shaft.

**Figure 2:**
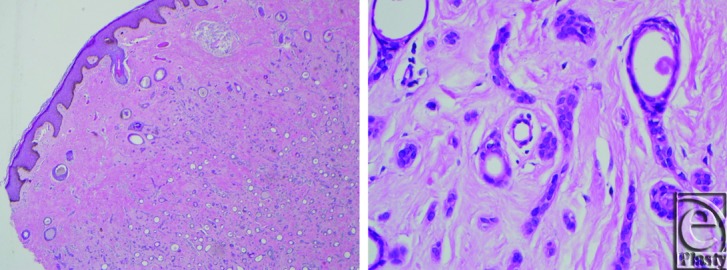
Biopsy specimen demonstrating typical syringoma features. (*Left*) Basal hyperpigmentation in the epidermis (Hematoxylin-eosin stain; original magnification × 4). (*Right*) Characteristic dermal coma-shaped structures lined by 2 rows of epithelial cells surrounded by collagen bundles, with colloidal material within the lumina (Hematoxylin-eosin stain; original magnification × 40).

A 29-year-old man presented with a 5-year history of multiple asymptomatic lesions of the penis. The patient had no significant medical history and sexually transmitted infections were ruled out. Physical examination demonstrated multiple clustered skin-colored papules along the dorsum and sides of the penis, extending from the base to approximately two thirds the length of the penile shaft (Fig 1). Punch biopsy demonstrated multiple syringomas (Fig 2). The patient was significantly bothered by the appearance of these lesions and was offered surgical excision and reconstruction.

## RESULTS

**Figure 3:**
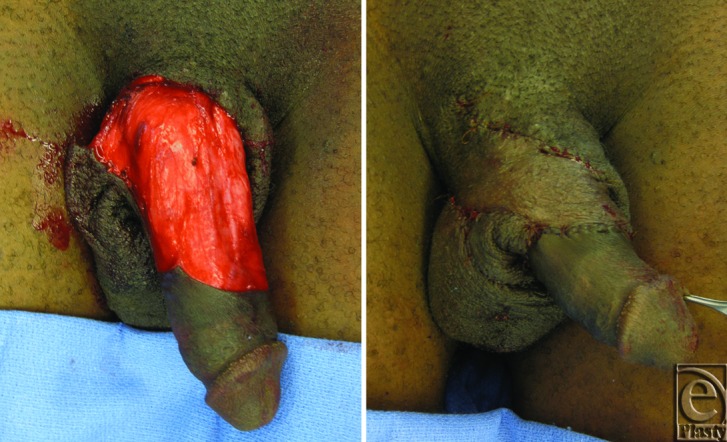
(*Left*) Penile defect after skin resection superficial to Buck's fascia. (*Right*) Reconstruction of penile defect with scrotal skin flaps.

Involved skin was resected superficial to Buck's fascia (Fig 3). A scrotal skin flap was then designed, elevated, and transposed into position across the dorsal defect. The donor site defect on the ventral aspect of the penis was resurfaced with additional scrotal skin flaps elevated in the same plane over distance of 25 × 20 cm. 3-0 Vicryl deep sutures and 4-0 chromic skin sutures were used for flap insetting and donor site closure.

**Figure 4:**
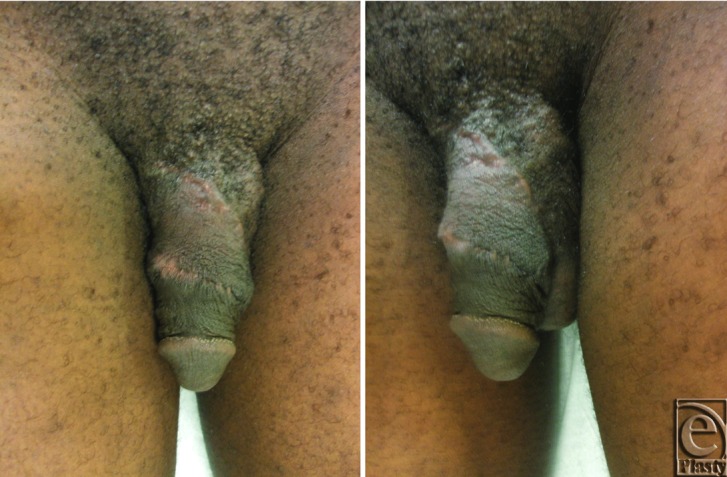
Follow-up images of penile reconstruction 130 days postoperatively.

The postoperative course was unremarkable, and the patient was discharged the following day. He has remained very satisfied with his postoperative result (Fig 4). He has some minor firmness over the scar line on the penile shaft, but has no chordee, can maintain erections, and is sexually active.

## DISCUSSION

To our knowledge, this represents the 12th reported case of penile syringoma and the first described surgical excision and reconstruction of multiple penile syringomas. The differential diagnosis for penile syringoma is extensive and partly includes bowenoid papulosis, sebaceous hyperplasia, epidermoid cysts, lichen planus, hamartomas, sarcoidosis, angiofibromas, and genital warts.^8^^,^^9^^,^^17^ Because of their location, patients are usually distressed due to concern of a sexually transmitted infection and/or the appearance of such to their partners.^15^

Treatment is necessary only for cosmetic reasons. Apart from surgical excision, several other treatment modalities exist including electrocoagulation, liquid nitrogen cryotherapy, dermabrasion, and CO2 lasers.^15^ However, given the intradermal location of these eccrine neoplasms, complete removal by these methods is difficult and reoccurrence is common.^18^

Surgical excision allows for the complete removal of the lesion but is more invasive and results in scarring. In the majority of instances, which are usually isolated, surgical excision is unnecessary. If the patient desires excision of isolated syringomas, closure can be achieved with elliptical excision, local undermining, and linear closure.

In our patient, multiple lesions were present over a large area of the shaft, necessitating skin grafting or local tissue rearrangement for coverage. We found scrotal flaps to be a viable option for reconstruction. Pliable adjacent scrotal tissue allows for tension-free closure of large defects and is an excellent functional substitute for penile skin as the loose connective tissue within scrotal dartos fascia permits adaptive conformation to penile spongiform changes. In addition, scrotal tissue possesses a rich blood supply via paired posterior scrotal arteries (superficial vessels from the deep internal pudendal arteries) lying within the dartos fascia, resulting in a robust vascular flap. Although reconstruction of the penile shaft with scrotal flaps carries some notable drawbacks, including transfer of hair follicles, sebaceous glands, and skin with dissimilar pigmentation, we do not think this functional result would have been achievable with other reconstructive options.

## CONCLUSIONS

When faced with reconstructing the penile shaft after excision of multiple syringomas, benefits and drawbacks of all reconstructive options should be discussed with patients. Scrotal flaps should be considered in the reconstructive armamentarium to maximize penile functionality through normalization of shaft alignment and maintenance of erectile function.
